# Hybrid treatment of an unusual traumatic aortic arch rupture with pseudoaneurysm: a case report

**DOI:** 10.1186/s13019-019-0896-9

**Published:** 2019-04-08

**Authors:** Shoujun Tang, Shengjie Tang, Li Yu, Yongheng Zhang, Haining Zhou

**Affiliations:** 1 Cardiovascular Center, Suining Central Hospital, No.127, West Desheng Road, Chuanshan District, Suining, 629000 Sichuan China; 2 Department of ECG, Suining Central Hospital, Suining, 629000 China

**Keywords:** Hybrid treatment, Traumatic, Pseudoaneurysm, Thoracic endovascular aortic repair (TEVAR)

## Abstract

**Background:**

Traumatic aortic arch rupture with pseudoaneurysm is a rare high fatal injury. Thoracic endovascular aortic repair(TEVAR) is widely used in the treatment of aortic diseases. However a unified traumatic aortic injury risk stratification tool still lacks. The patients’ disease assessment and operation method still rely on the doctors’ subjective anticipation of disease and surgical results.

**Case presentation:**

A 31-year-old male presented with chest pain and hoarseness of recent onset. Imageological examination showed aortic rupture with mediastinal pseudoaneurysm in Zone 1. Debranching + TEVAR hybrid operation was considered: Using artificial blood vessels rebuild the arch branches, the covered stent was placed in aortic arch for endovascular repair via femoral artery. The follow-up was good in postoperative 7d, 1 year and > 2 years.

**Conclusion:**

The traumatic aortic arch rupture with pseudoaneurysm treated by Debranching+TEVAR hybrid operation is feasible. The short-term and medium-term follow-up results are satisfactory. For traumatic aortic arch injury, hybrid operation is recommended to reduce the risk of cardiopulmonary bypass and deep hypothermia circulatory arrest under no open operation conditions or emergency situation.

## Background

Blunt traumatic aortic injury (BTAI) is derived from the blunt injury due to decelerated impact. It is common in traffic accident and falling. The annual incidence rate is estimated 20–30/million in the United States, accounting for 0.3% of traumatic events [1]. In 1958, Parmley reported that the pre-hospital mortality of BTAI was 85%. With the increase of the pre-hospital first-aid level, the pre-hospital mortality of these patients was decreased to 54–80%. The mortality was 50% after admission for 24 h and continued to be the second fatal cause of traumatic patients [2]. The very low morbidity and high mortality make patients difficultly survive and be admitted in hospital.

### Case presentation

**Fig. 1:**
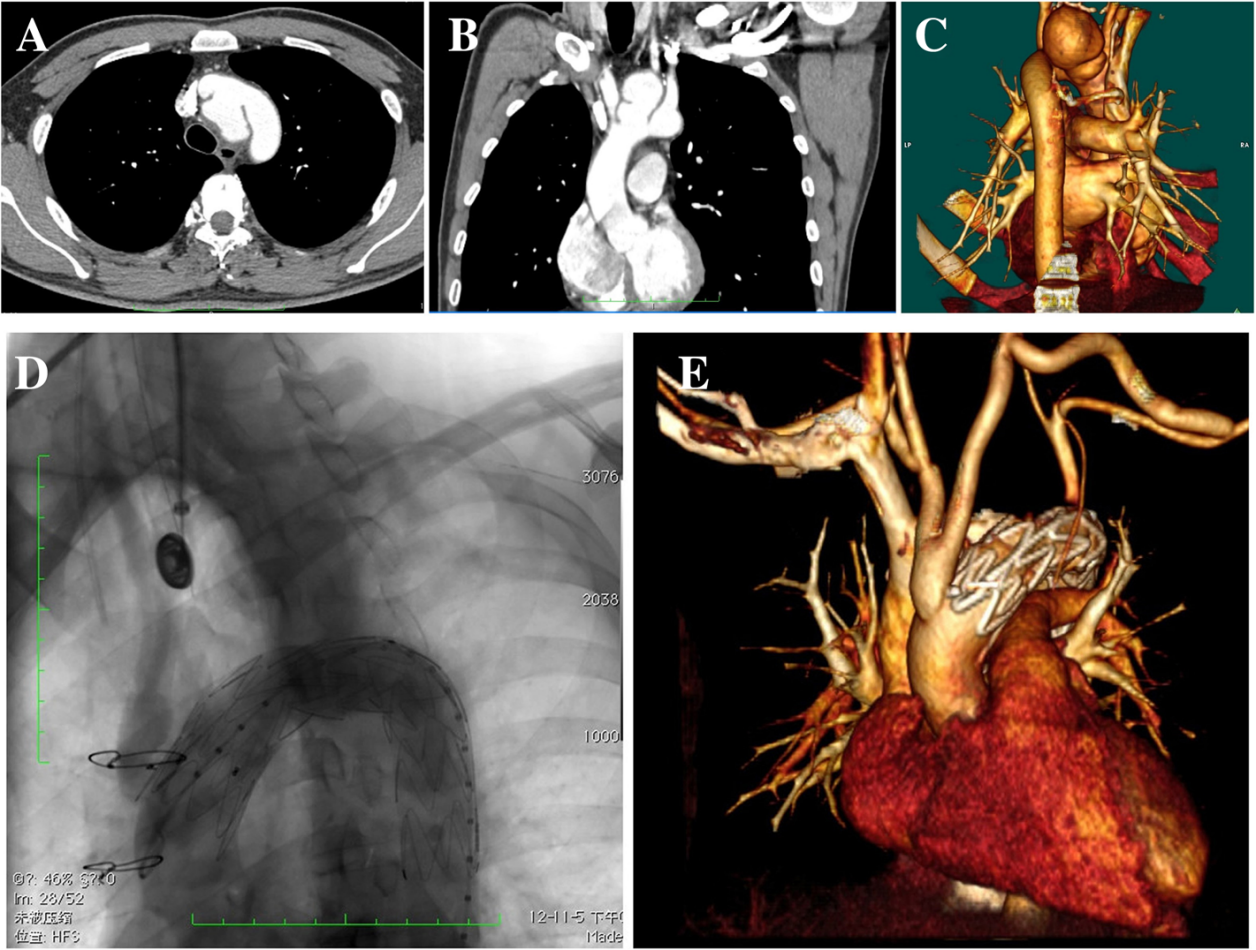
(**a**-**c**) Preoperative CT; (**d**) Intraoperative angiography; (**e**) CT angiography on the 7th postoperative day

A 31-year-old male presented with chest pain and hoarseness of recent onset. The patient had suffered a traffic accident 3 months before admission, resulting with femur, radius, ribs and sternum fractures; and had undergone internal fixation of the femur and radius. The physical examination was normal. Computed tomography (CT) showed a ruptured aortic arch with pseudoaneurysm. The crevasse measured 20 mm and was located in the posteroinferior aspect of the aortic arch, involving the posterior wall of the innominate artery and the origin of left common carotid artery. The pseudoaneurysm of 40 mm × 48 mm × 30 mm was located in the upper mediastinum. The distance from the junction of aortic sinus and ascending aorta to the rupture was 7.9 cm (Figs. 1 a-c). The recent appearance of symptoms suggested that the pseudoaneurysm had increased rapidly and compressed the left recurrent laryngeal nerve. In this case, to reduce the risk of cardiopulmonary bypass or cardiac arrest under deep hypothermia [3], a hybrid operation was performed: debranching + thoracic endovascular aortic repair (TEVAR). The involved branches of the aortic arch were rebuilt with artificial vessels, and a covered stent was placed in the aortic arch for endovascular repair via femoral artery. A hybrid operation is safer, more feasible, and more comprehensive than other treatments for some high-risk patients. Thoracic and abdominal CT angiography performed on the 7th postoperative day showed that the contrast agent did not leak and that the reconstruction of blood vessels was smooth (Figs. 1 d and e). Recovery was uneventful.

## Discussion

Blunt traumatic aortic injury is a rare high fatal decelerated injury. Its symptoms are obvious but signs are not obvious. The injury part is usually located in the aortic isthmus (54–65%), rare in the ascending aorta at the proximal end of the innominate artery (10–20%), aortic arch and descending aorta [4]. Once aortic aneurysm was missed or early expansion was not obvious, it was difficult to find and the accidental death much more likely occur. A few patients without symptoms were diagnosed by accident examination or chest and back pain, superior vena cava syndrome, hoarseness, dysphagia, cough, hemoptysis and other symptoms occurred. This kind of symptoms often suggested that the tumor body was increased and the disease was evolved. Once the diagnosis was confirmed, the timely surgical repair should be performed.

Endovascular repair is widely used in the treatment of aortic diseases. The Debranching endovascular repair achieved satisfactory short-term and mid-term results in aortic arch injury. The Hybrid operation mode dose not require cardiopulmonary bypass or deep hypothermia circulatory arrest, which provides a less invasive and treatment of injury of aortic arch [5].

The rupture position, anchoring region Debranching involvement and aortic arch curvature require to be carefully evaluated to prevent the “bird beaking” after stent implantation, and then to increase the endoleak rate even aortic rupture. The sequential clipping order of Debranching required to be evaluated, through CTA or MRA or digital subtraction angiography for the cerebral blood vessels. In this case, the patient was the left vertebral artery dominant. Debranchings were clipped and anastomosed from right to left. The left common carotid artery-branch vessels end-to-end anastomosis and left common carotid artery-left subclavian artery bypass anastomosis were performed on the neck incision and supraclavicular incision. After the left common carotid artery was clipped, the anastomosis of the two could be performed at the same time, so as to reduce the occlusion time of left common carotid artery. In order not to block the left vertebral artery, left subclavian artery anastomosis was performed in the distal end of initial left vertebral artery. The length of the proximal landing zone of the aortic arch was recommended for at least 20 mm. The diameter of the stent was larger than 10–15% of the landing zone, so as to avoid the type I endoleak. After the left common carotid artery-left supraclavicular bypass was completed, the proximal end of the left clavicle artery was ligated or closed with a coil or plug via the left radial artery, to prevent type II endoleaks.

## Conclusions

Traumatic pseudoaneurysm is a serious complication of local vascular injury. Hybrid debranching technique greatly reduces surgical trauma and provides satisfactory outcome and good function recovery. It is safer, more feasible, and more comprehensive than other treatments for some high-risk patients.

## Abbreviations

BTAI: Blunt traumatic aortic injury
CT: computed tomography
TEVAR: thoracic endovascular aortic repair
